# The Large Right Heart Is Associated with the Prolongation of the Procedure Time of Leadless Pacemaker Implantation

**DOI:** 10.3390/medicina57070685

**Published:** 2021-07-04

**Authors:** Naoya Kataoka, Teruhiko Imamura, Takahisa Koi, Hiroshi Ueno, Koichiro Kinugawa

**Affiliations:** Second Department of Internal Medicine, University of Toyama, Toyama 930-0194, Japan; nkataoka@med.u-toyama.ac.jp (N.K.); taka1010@med.u-toyama.ac.jp (T.K.); hueno@med.u-toyama.ac.jp (H.U.); kinugawa@med.u-toyama.ac.jp (K.K.)

**Keywords:** Micra, procedure time, right heart

## Abstract

*Background and objectives:* Leadless pacemakers are less invasive but are as effective as conventional pacemakers and are increasingly implanted in elderly patients. However, the implantation procedure is sometimes challenging in patients with abnormal anatomy, particularly those with an enlarged right heart. We aimed to determine the right heart parameters that were associated with longer procedure times for leadless pacemaker implantation. *Materials and Methods:* Among 19 consecutive patients in whom Micra leadless pacemakers (Micra TPS, Medtronic, Minneapolis, MN) were implanted, the diameter and area of both the right atrium and right ventricle were measured by transthoracic echocardiography before the procedure. The right heart parameters that were associated with a procedure time > 60 min were investigated. *Results*: In the 19 patients (median 81 years old, 10 male) who underwent implantation of the Micra system, 6 (32%) required a procedure time > 60 min. Among the baseline right heart echocardiographic parameters, right atrial diameter and area were significantly associated with a procedure time > 60 min (odds ratio 11.3, 95% confidence interval 1.09–1.17, *p* = 0.042; and odds ratio 1.57, 95% confidence interval 1.05–2.34, *p* = 0.029, respectively) at a cutoff of 4.0 cm and 17.0 cm^2^, respectively. *Conclusions:* Patients with an enlarged right atrium may not be good candidates for leadless pacemakers given the longer procedure time, and conventional pacemakers should perhaps be recommended as an alternative.

## 1. Introduction

The Micra system (Micra TPS, Medtronic, Minneapolis, MN), which is the first leadless pacemaker, is a lead- and pocket-less implantable device having a high successful implantation rate (99.2%), a low incidence of complications (4%), and a 93.3% 6-month freedom from serious adverse events [1,2,3,4]. The post-approval registry also demonstrates similar favorable outcomes in terms of implantation and complications [5].

Recently, we reported a patient with a greatly enlarged right atrium accompanied by atrial septal deficiency in whom a leadless pacemaker failed to be implanted [6]. Instead, a conventional pacemaker was successfully implanted via the subclavian vein. Another team also reported a challenging case for leadless pacemaker implantation with an enlarged right heart [7]. However, there have been few studies investigating right heart anatomical parameters associating with longer procedure time. Such data would be useful in determining the optimal therapeutic strategy, i.e., a Micra system or conventional pacemaker, for each patient. The present study aimed to investigate echocardiographic parameters associating with a longer procedure time for implanting the Micra leadless pacemaker.

## 2. Materials and Methods

### 2.1. Study Population

Consecutive patients who underwent implantation of the Micra system successfully at our institute between August 2019 and March 2021 were included retrospectively. All patients had bradycardia due to atrial fibrillation, sick sinus syndrome, or advanced atrioventricular block. The present study was approved by the institutional review board at the University of Toyama (R2020025 approved on 1 May 2020). Informed consent was obtained from all patients.

### 2.2. Baseline Clinical Characteristics

Baseline clinical characteristics, including demographics, comorbidity, and laboratory data, were retrieved from the electronic medical record.

### 2.3. Right Heart Parameters and Procedure Times

Echocardiographic parameters including right atrial diameter, right atrial area, right ventricular end-diastolic area, right ventricular basal diameter, and right ventricular mid diameter were measured according to the current guideline using the four-chamber views focusing on the right heart by an expert cardiologist blinded to the study data within 30 days before Micra implantation [8].

The procedure time for the implantation was defined as a time from the infusion of local anesthesia to the sheath withdrawal. A procedure time > 60 min was defined as the primary endpoint.

### 2.4. Statistical Analysis

Data were expressed as median (interquartile range) and compared between the groups using the Mann–Whitney U test. Categorical data were expressed as numbers (percentages) and compared using Fischer’s exact test. Univariable logistic regression analyses were performed to investigate echocardiographic right heart parameters associating with the procedure time > 60 min. Receiver operating characteristics analyses were performed to calculate cutoffs to predict a procedure time > 60 min. A two-sided *p* value < 0.05 was considered as statistically significant. Data analysis was performed using SPSS Statistics 22 (SPSS Inc, Armonk, IL, USA).

## 3. Results

### 3.1. Patient Characteristics

Nineteen patients (median 81 years old, 10 male) were included (Table 1). Of them, 6 (32%) were in the procedure time > 60 min group. Age, sex, body mass index, atrial fibrillation indication, hypertension, chronic obstructive pulmonary disease, and dialysis, all of which have been reported as risk factors for Micra system-associated complications, were statistically not significantly different between the two groups stratified by the procedure time [3]. However, both atrial and ventricular parameters were significantly higher in the group with procedure time > 60 min than in the group with procedure time ≤ 60 min in the univariable analysis.

### 3.2. Right Heart Parameters and the Procedure Time

**Figure 1:**
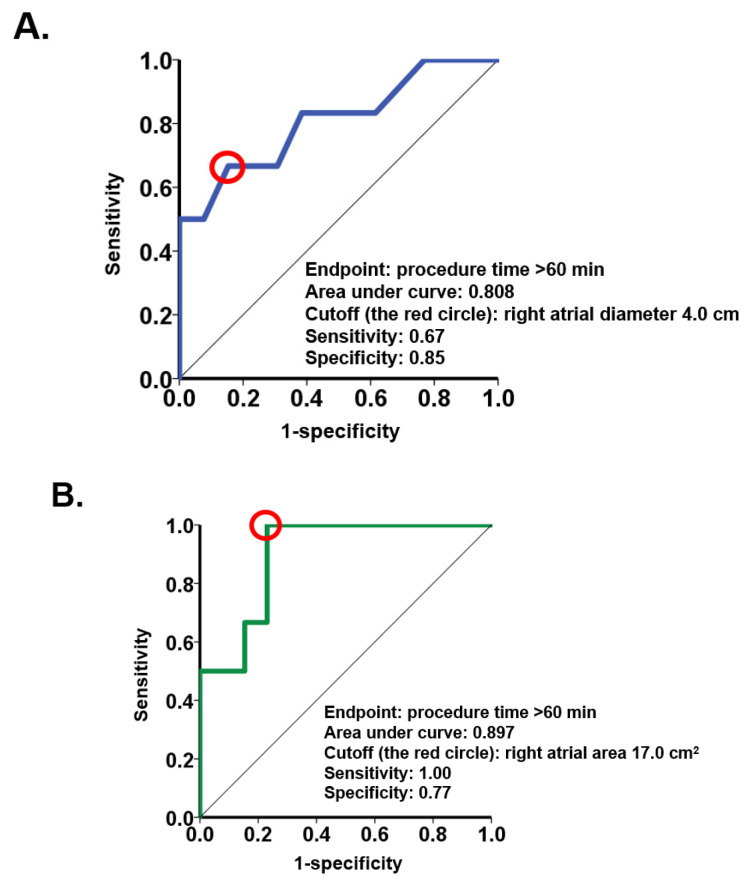
Receiver-operating characteristic curve for predicting procedure time > 60 min. (**A**) Receiver-operating characteristic curve of right atrial diameter. (**B**) Receiver-operating characteristic curve of right atrial area.

Echocardiographic right heart parameters obtained before the procedures were analyzed in the logistic regression. Baseline right atrial diameter and right atrial area were associated with procedure time > 60 min with an odds ratio of 11.3 (95% confidence interval 1.09–117; *p* = 0.042) for right atrial diameter and 1.57 (95% confidence interval 1.05–2.34; *p* = 0.029) for right atrial area (Table 2). The parameters of the right ventricle failed to show significance. ROC analyses demonstrated a cutoff of 4.0 cm for right atrial diameter and a cutoff of 17.0 cm^2^ for right atrial area to predict procedure time >60 min (Figure 1).

**Figure 2:**
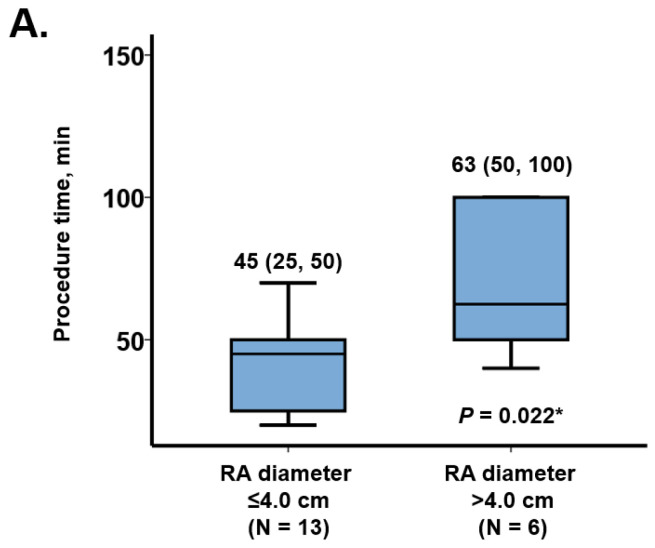

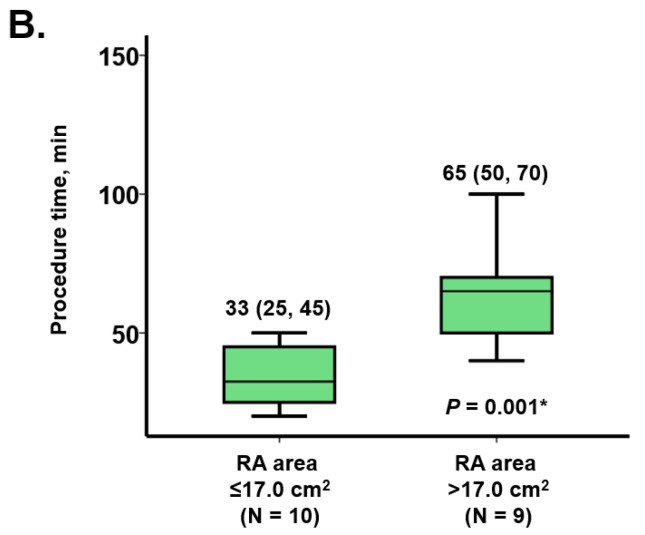
Comparison of procedure time between the two groups according to the cutoff that best discriminates procedure time > 60 min. (**A**) Right atrial diameter; (**B**) right atrial area. RA = right atrium. * Means significant difference.

Procedure time was significantly longer in the patients with right atrial diameter above the cutoff (63 (50, 100) minutes versus 42 (25, 50) minutes, *p* = 0.022; Figure 2A). In the same manner, patients with right atrial area above the cutoff had longer procedure time (65 (50, 70) minutes versus 33 (25, 45) minutes, *p* = 0.001; Figure 2B).

**Figure 3:**
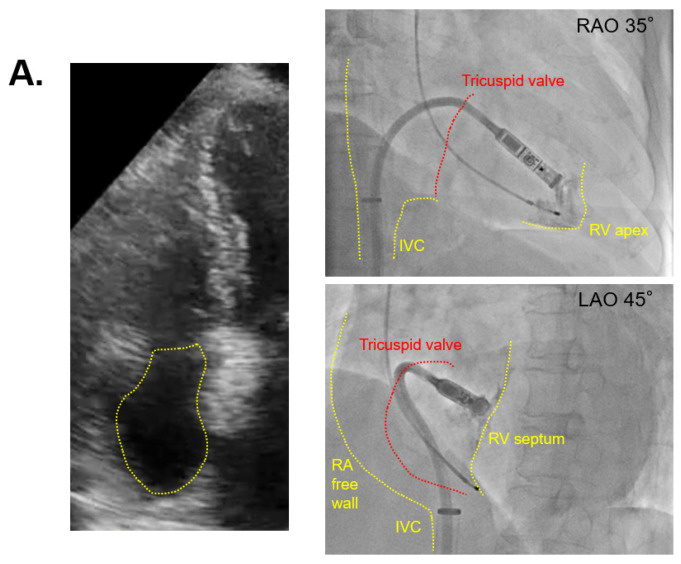

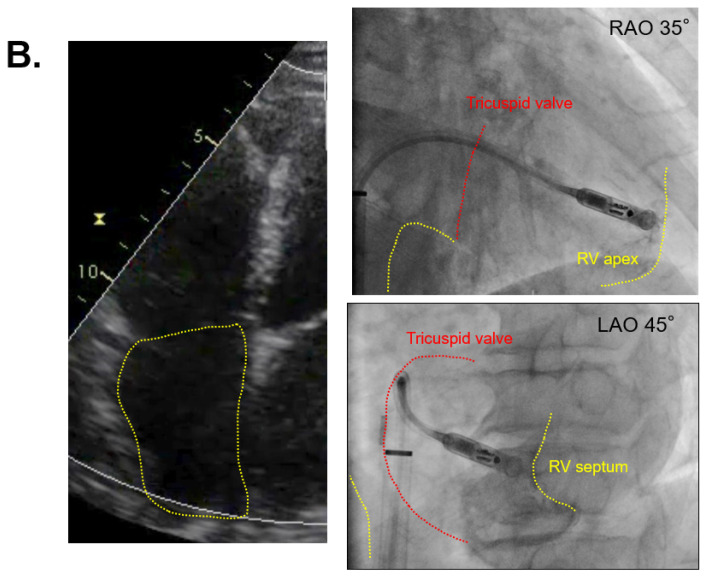
Echocardiography in the shortest and longest procedures. (**A**) The case of smaller right atrium with 30 min of procedure time; (**B**) the case of larger right atrium with 185 min of procedure time; echocardiographic views indicate four chambers, viewing from the apex, and yellow dashed lines indicate the right atrium; IVC = inferior vena cava, RV = right ventricle.

The two cases with the shortest and longest procedure times are displayed in Figure 3A,B, respectively. The patient of Figure 3A had a smaller right atrium with 3.3 cm right atrial diameter and 14.7 cm^2^ right atrial area, and a procedure time of 30 min. The patient of Figure 3B had a larger right atrium with 4.3 cm right atrial diameter and 24.7 cm^2^ right atrial area, and the procedure time was 185 min (Figure 3B).

## 4. Discussion

This is the first study demonstrating the association between right atrial size and procedure time for implanting the Micra leadless pacemaker.

## 5. Previously Reported Risk Factors of Procedure-Related Complications

Several baseline characteristics, including body mass index < 20 kg/m^2^, age ≥ 85 years, female sex, non-atrial fibrillation indication, and chronic lung disease, were reported as the risk factors for procedure-related complications, such as perforation and pericardial effusion [9]. Detailed explanations are unknown, but these parameters may be associated with a small heart cavity that hinders safe catheter procedure and increases the risk of cardiac injury. In this study, we had no such critical complications, probably due to the very low incidence rate of these complications in general.

However, we sometimes experience Micra implantation procedures that required >60 min. A longer procedure time would be associated with minor procedure-related troubles, which might result in critical complications. A longer procedure time would also be associated with more radiation exposure. We previously experienced a case in which Micra implantation failed with a prolonged procedure time, but a conventional pacemaker was easily implanted via the subclavian vein. Factors associated with a longer procedure time would be favor choice of a conventional pacemaker over the Micra system beforehand. This is why we investigated factors associating with longer procedure time. We defined >60 min of procedure time as a challenging procedure, considering that 45 ± 15 min is the average procedure time for Micra implantation according to a previous paper [10].

## 6. The Implications of Enlarged Right Heart for the Micra Implantation

A previous case report showed that a patient with an enlarged right heart due to a rheumatic fever required a unique technique to deliver the Micra system [7]. Our case report also presented a greatly enlarged right heart due to an atrial septum defect leading to failed Micra implantation [6]. However, there have been few studies investigating the association between cardiac anatomical features and difficulty in Micra implantation.

**Figure 4:**
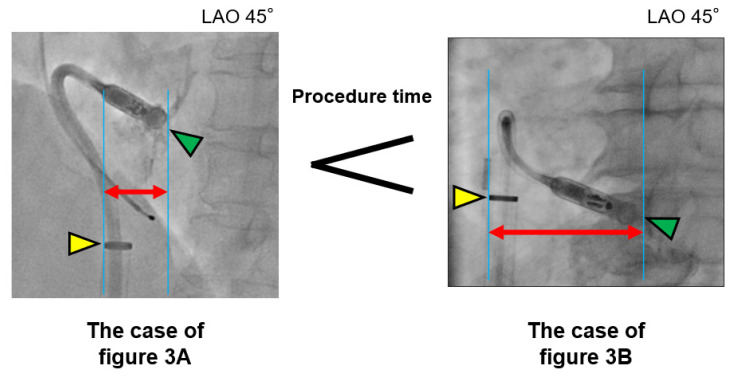
Schematic images of right atrium size and catheter push to the right ventricular septum. The delivery sheath in a larger right atrium (**right side**) may be far away from the right ventricular septum compared with that in a smaller right atrium (**left side**). Yellow triangles indicate the end of the sheath; green triangles, the edge of the Micra delivery system; and red arrows, the length from the right ventricular septum to the sheath. The scale magnification is the same between the two images.

The Micra system should tightly contact the right ventricular septum to make a “gooseneck sign”. In patients with an enlarged right atrium, the delivery sheath may be far away from the right ventricular septum, making it difficult to contact the septum tightly compared to patients with a small right atrium (Figure 4). This may be why right atrial size is associated with lengthened procedure times and not ventricular features.

We proposed cutoffs of right atrial diameter and area at 4.0 cm and 17 cm^2^, respectively. When a patient’s right atrium reaches one of these cutoffs, the Micra implantation may be challenging, and we recommend conventional pacemaker implantation via the subclavian vein. Micra implantation via jugular vein is a feasible alternative, although specific device and implant techniques are required [11].

## 7. Study Limitations

The present study has several limitations. This is a single-center retrospective observational study with a small sample size; further large-scale studies are warranted. The parameters associated with procedure time > 60 min were detected by univariable but not multivariable analysis, probably due to the small sample size. We included standard parameters to assess right heart size, but other unique parameters, for example, tricuspid valve regurgitation or pulmonary artery hypertension may exist to show the anatomical and functional features of right heart. The prognostic impacts of the prolonged procedure time should be assessed in further prospective studies. Finally, the proficiency of the operators, who were two board-certificated attending doctors in this study, or the length of vascular access procedure may affect the total procedure time.

## 8. Conclusions

Although larger-scale studies are needed, the diameter and area of the right atrium were associated with a Micra system implantation procedure time > 60 min in this study.

## Figures and Tables

**Table 1:** Baseline characteristics.

	Total (N = 19)	Operation Time >60 min (N = 6)	Operation Time ≤60 min (N = 13)	*p* Value
Demographics				
Age, years	81 (77, 89)	81 (76, 91)	81 (77, 87)	1.0
Male	10 (53%)	4 (67%)	6 (46%)	0.37
Body mass index	22.6 (18.9, 24.8)	22.6 (18.9, 24.8)	22.6 (19.2, 23.8)	0.97
Comorbidity				
Diabetes mellitus	1 (5%)	0	1 (8%)	0.68
Hypertension	13 (68%)	5 (83%)	8 (62%)	0.35
Dyslipidemia	6 (32%)	1 (17%)	5 (38%)	0.35
Chronic obstructive pulmonary disease	1 (5%)	0	1 (8%)	0.68
Ischemic heart disease	8 (42%)	3 (50%)	5 (38%)	0.51
Hemodialysis	2 (11%)	1 (17%)	1 (8%)	0.91
Laboratory data				
Hemoglobin, g/dL	11.7 (10.7, 14.0)	12.6 (11.1, 14.1)	11.7 (10.7, 12.9)	0.52
eGFR, mL/min/1.73 m^2^	33.0 (18.0, 41.7)	28.5 (17.5, 35.6)	33.0 (26.5, 43.8)	0.52
Plasma B-type natriuretic peptide, pg/mL	179 (67, 414)	223 (162, 274)	165 (67, 414)	1.0
Indications				
Atrial fibrillation	7 (37%)	4 (67%)	3 (23%)	0.13
Sick sinus syndrome (Rubenstein III)	8 (42%)	2 (33%)	6 (46%)	0.60
Advanced atrioventricular block	4 (21%)	0	4 (31%)	0.26
Right heart echocardiographic data				
Right atrial diameter, cm	3.4 (3.2, 4.1)	4.2 (3.4, 4.5)	3.3 (3.2, 3.8)	0.036 *
Right atrial area, cm^2^	16.1 (11.6, 21.6)	21.7 (19.5, 23.6)	13.5 (11.5, 16.1)	0.005 *
Right ventricular end-diastolic area, cm^2^	17.8 (12.7, 19.2)	19.6 (18.7, 21.2)	14.5 (12.2, 17.8)	0.001 *
Right ventricular basal diameter, cm	3.5 (3.3, 4.1)	3.9 (3.5, 4.6)	3.4 (3.2, 3.8)	0.046 *
Right ventricular mid diameter, cm	3.1 (2.9, 3.5)	3.5 (3.0, 3.6)	2.9 (2.6, 3.3)	0.072
Procedure time, min	45 (30, 65)	68 (64, 121)	40 (25, 48)	<0.001 *

eGFR, estimated glomerular filtration ratio. Continuous variables were expressed as median and interquartile and compared between the groups using Mann–Whitney U test. Categorical variables were expressed as number and percentage and compared between the groups using Fischer’s exact test. * *p* < 0.05.

**Table 2:** Echocardiographic right heart parameters associated with procedure time > 60 min.

	Odds Ratio (95% Confidence Interval)	*p* Value
Right atrial diameter, cm	11.3 (1.09–117)	0.042 *
Right atrial area, cm^2^	1.57 (1.05–2.34)	0.029 *
Right ventricular end-diastolic area, cm^2^	2.90 (0.84–9.98)	0.091
Right ventricular basal diameter, cm	4.42 (0.71–27.4)	0.11
Right ventricular mid diameter, cm	14.1 (0.67–300)	0.089

Variables significantly different in the comparison analyses were included. * *p* < 0.05 by logistic regression analyses.
